# A historical Hawaiian *Avipoxvirus* genome reconstructed from an 1898 museum specimen

**DOI:** 10.1016/j.isci.2025.112153

**Published:** 2025-03-03

**Authors:** Madeline W. Eibner-Gebhardt, Robert C. Fleischer, Michael G. Campana

**Affiliations:** 1 Center for Conservation Genomics, Smithsonian’s National Zoo and Conservation Biology Institute, Washington, DC, USA; 2 School Without Walls, Washington, DC, USA; 3 The College of William & Mary, Williamsburg, VA, USA; 4 George Mason University, Fairfax, VA, USA

**Keywords:** Virology, Genomics, Phylogenetics

## Abstract

*Avipoxvirus* is an avian pathogen that likely contributed to the declines and extinctions of endemic Hawaiian birds since its 19th century introduction. We surveyed 719 DNA libraries, including 639 representing 440 Hawaiian bird specimens, for evidence of *Avipoxvirus* infection. We reconstructed a 5.2× *Avipoxvirus* genome from an 1898 Hawaii ‘amakihi (*Chlorodrepanis virens*) specimen. Its sequence matched an extant Hawaiian *Avipoxvirus* strain, supporting the strain’s persistence in Hawaii over the last century. We identified the earliest molecularly verified case of *Avipoxvirus* in the Hawaiian Islands in an 1887 ʻalalā (*Corvus hawaiiensis*) specimen and reconstructed a partial *Avipoxvirus* genome from this specimen. Both specimens’ *Avipoxvirus* strains were most closely related to canarypox virus, suggesting that introduced passerines may be the source of *Avipoxvirus* in Hawaiian endemic land birds. These findings clarify the origins and evolution of *Avipoxvirus* in Hawaii and provide evidence for the broader role of pathogens in driving biodiversity loss.

## Highlights

• *Avipoxvirus* contributed to 19th century Hawaiian bird die-offs
• Earliest verified Hawaiian *Avipoxvirus* case found in an 1887 ‘alalā museum specimen
• We reconstruct a complete Hawaiian *Avipoxvirus* genome from 1898
• Hawaiian *Avipoxvirus* is most closely related to canarypox virus

## Graphical abstract

**Figure:**
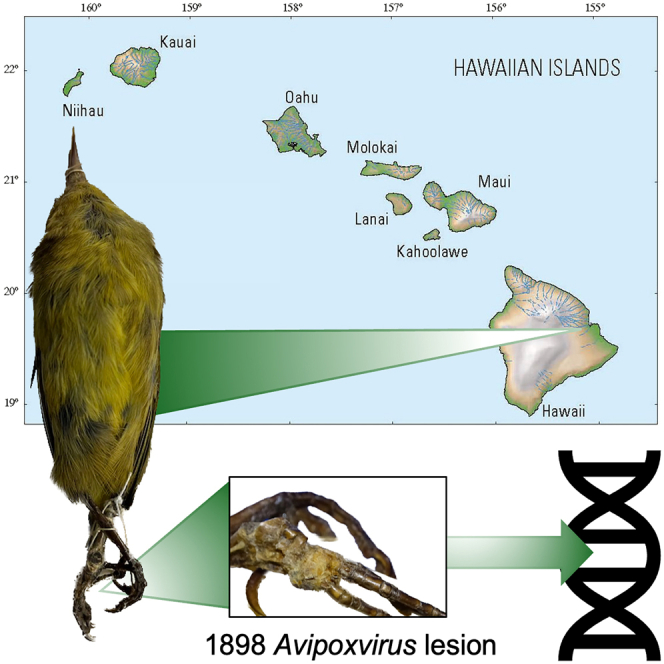

## Introduction

*Avipoxvirus* and avian malaria (*Plasmodium relictum*), two introduced pathogens vectored by the introduced mosquito *Culex quinquefasciatus*, are thought to have contributed to the decline and extinction of many endemic Hawaiian bird species since the mid-19th and early 20th century, respectively.^1^^,^^2^^,^^3^^,^^4^^,^^5^^,^^6^ These pathogens are among a variety of factors, including habitat loss and introduced predators, implicated in the extinctions of about two-thirds of Hawaii’s ∼108 endemic land bird species.^7^ Supporting *Avipoxvirus*’s continued presence and contribution to biodiversity loss in the islands, genetic and visual evidence has revealed historical *Avipoxvirus* infections, including that of a 1900 Hawaii ʻelepaio (*Chasiempis sandwichensis*).^2^^,^^3^ *Avipoxvirus*-like lesions were documented in an 1888 specimen of the extinct ‘ō‘ū (*Psittirostra psittacea*),^8^ and the extinct-in-the-wild ‘alala are known hosts of *Avipoxvirus*,^2^ suggesting that the pathogen may play a role in these species’ declines. Both *Avipoxvirus* and avian malaria continue to threaten the Hawaiian avifauna, known for its impressive radiations of phenotypic diversity.^9^ The significant impact of avian malaria in ongoing declines in extant Hawaiian endemics is well documented (most notably and more recently in Kauai^10^), but the role of *Avipoxvirus* in historical and ongoing extinctions is more equivocal due to lack of definitive evidence.^3^

While more research has focused on the history of avian malaria and its effects upon Hawaiian avifauna^3^^,^^4^^,^^5^, *Avipoxviruses*—and Hawaiian *Avipoxviruses* in particular—remain understudied.^3^ *Avipoxviruses* cause damaging lesions on the feet and around the beak and eyes. *Avipoxvirus* infection can cause mortality in Hawaiian endemic land birds in laboratory settings, but is a less significant driver of mortality in extant wild Hawaiian land birds compared to avian malaria.^3^ However, this does not preclude the suggested major role for *Avipoxvirus* in the late 19th century Hawaiian avifauna declines and extinctions as extant individuals may have adapted to the invasive pathogen over the last century (survivorship bias),^3^^,^^6^ or *Avipoxvirus* may have evolved lower virulence. Previous genetic analyses based on the *4b core protein* gene found at least 2 Hawaiian *Avipoxvirus* strain clusters in Hawaiian land bird species that vary in virulence and that Hawaiian cluster 2 had persisted in the islands since at least 1900.^2^ Both strain clusters are part of *Avipoxvirus* subclade B1,^11^ which contains canarypox viruses and primarily infects passerines, often with high mortality.^2^^,^^12^ These lineages have been recovered from at least 8 Hawaiian species, including at least 6 endemics and 2 exotics.^2^ A third lineage identical to fowlpox virus (commonly found in poultry) and a fourth lineage appearing to match pigeonpox virus have also been identified in the Hawaiian Islands.^3^^,^^13^

Previous studies have recovered genetic evidence of *Avipoxviruses* from historical museum specimens from Hawaii,^2^ the Galapagos Islands,^14^ Spain,^11^ Peru,^11^ and Japan.^11^ These studies were limited to amplifying short regions (up to 538 bp) of the *4b core protein* gene, limiting these analyses to pathogen detection and strain classification. No genome of the Hawaiian canarypox-like *Avipoxvirus* strains has yet been sequenced.

To clarify the origins and evolution of Hawaiian *Avipoxviruses*, we screened sequences from a total of 639 DNA Illumina sequencing libraries derived from 440 Hawaiian bird specimens (extant blood samples and museum tissues) for evidence of *Avipoxvirus* DNA. Samples were collected and sequenced as part of other ongoing projects (STAR Methods). Screened samples consisted of a mix of shotgun-sequenced and hybridization-captured libraries.

## Results

### Screening results

Seven libraries yielded *Avipoxvirus* DNA sequences (Table S1). Two museum specimens—USNM 169333 and CamMZ-27/Cor/5/gg/8—yielded 27,172 and 1,146 sequences, respectively. USNM 169333 is a Hawaii ʻamakihi collected in 1898 in Kaumana, Hawaii Island. CamMZ-27/Cor/5/gg/8 is an ʻalalā collected in 1887 in Kaawaloa, Kona, Hawaii Island. Five libraries yielded between 1 and 23 *Avipoxvirus* sequences, precluding further analysis due to low coverage. All 7 positive libraries were derived from museum toe pads, where *Avipoxvirus* is likely to accumulate in lesions. Furthermore, both USNM 169333 and CamMZ-27/Cor/5/gg/8 had visible evidence of pox lesions, supporting the *Avipoxvirus* DNA’s endogeneity. USNM 169333 and CamMZ-27/Cor/5/gg/8 had DNA fragmentation and cytosine deamination profiles consistent with century-old historic DNA^15^ (Figures S1 and S2). This indicates that we recovered true historical infections, rather than modern contamination. No blood sample yielded *Avipoxvirus* sequences, consistent with previous research that found *Avipoxviruses* to be difficult to detect in blood using genetic assays.^1^

### Phylogenetic analyses

USNM 169333 yielded sufficient *Avipoxvirus* sequences to reconstruct a near complete historical genome, while CamMZ-27/Cor/5/gg/8 yielded a partial genome. To determine the *Avipoxvirus* strains, we aligned the USNM 169333 and CamMZ-27/Cor/5/gg/8 *Avipoxvirus* reads against the reference genomes for divergent *Avipoxvirus* strains (canarypox virus: GenBank: NC_005309; fowlpox virus: GenBank: NC_002188; magpiepox virus: GenBank: MK903864; penguinpox virus 1: GenBank: NC_024446.1; and penguinpox virus 2: GenBank: MW296038). Nearly all reads for both specimens (USNM 169333: 27,005 reads [99.4%]; CamMZ-27/Cor/5/gg/8: 1,137 reads [99.2%]) mapped to canarypox virus, with decreasing numbers of reads mapping to reference genomes more genetically distant to canarypox virus (penguinpox virus 2: 25,684 reads in USNM 169333 and 1,065 reads in CamMZ-27/Cor/5/gg/8, respectively; magpiepox virus: 19,596 and 777; penguinpox virus 1: 764 and 19; and fowlpox virus: 541 and 17). The USNM 169333 genome sequence covered 98.1% (353,179 bp of 359,853 bp) of the canarypox virus genome reference and was sequenced to a mean depth of 5.2×, while CamMZ-27/Cor/5/gg/8 was sequenced to 17.1% coverage (61,872 sequenced bases). The novel USNM 169333 genome had a GC content of 30.4% and was 359,853 bp long, including 6,918 unresolved bases (6,641 unsequenced bases and 277 ambiguities) and 101 gaps compared to the canarypox virus genome. Visual inspection of the *Avipoxvirus* read alignments for both museum specimens showed even coverage of reads across the canarypox virus genome assembly for both samples, supporting the accuracy of the genomic reconstructions. The USNM 169333 genome sequence was 99.6% identical (351,688 identical sites) to canarypox virus, while the CamMZ-27/Cor/5/gg/8 was 99.4% identical (61,524 identical sites). Alignment of USNM 169333 and CamMZ-27/Cor/5/gg/8 *Avipoxvirus* sequences against Canarypox virus yielded a higher pairwise identity and number of identical sites than against other strains (penguinpox virus 2: 99.0% [332,140 identities] in USNM 169333 and 99.1% [57,258 identities] in CamMZ-27/Cor/5/gg/8; magpiepox virus: 96.8% [266,163 identities] and 97.8% [41,429 identities]; penguinpox virus 1: 89.5% [12,811 identities] and 95.0% [751 identities]; and fowlpox virus: 89.7% [9,994 identities] and 92.4% [775 identities). These results are consistent with previous research on the *Avipoxvirus 4b core protein* gene that found that Hawaiian *Avipoxvirus* strains were most similar to canarypox virus.^1^^,^^2^

**Figure 1:**
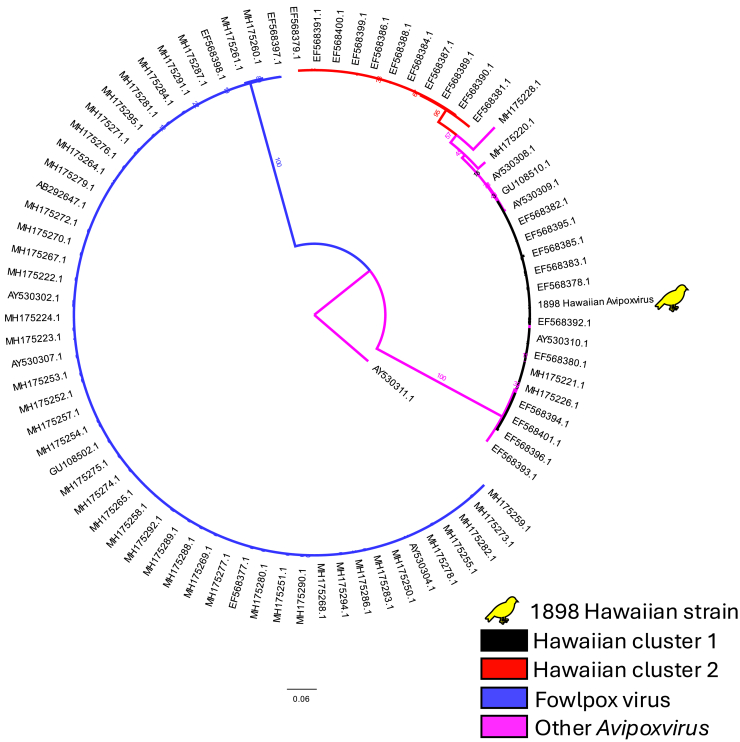
Maximum likelihood tree of *Avipoxvirus 4b core protein* gene sequences The newly reported 1898 Hawaiian *Avipoxvirus* strain falls within the Hawaiian canarypox-like cluster 1. Branch support is based on 100 bootstrap replicates. Scale bar denotes branch lengths in substitutions per site. Sequences are denoted by GenBank accession numbers.

We confirmed that the USNM 169333 *Avipoxvirus* strain was most similar to canarypox virus by alignment against 9 highly conserved genes^1^^,^^16^ (STAR Methods). The CamMZ-27/Cor/5/gg/8 genome was too fragmentary for reliable phylogenetic analysis using these conserved genes as we recovered only between 1 and 14 mapped reads per marker. Phylogenetic trees consistently clustered the USNM 169333 sequence with sequences from canarypox virus, shearwaterpox virus, and magpiepox virus (data available in the Smithsonian Figshare repository: https://doi.org/10.25573/data.25833037). The *4b core protein* sequence was identical to strain HAAM22.7H98 (GenBank: EF568392.1), a Hawaiian canarypox-like cluster 1 strain isolated from a Hawaii ‘amakihi sampled in 1998,^2^ supporting its persistence in Hawaii since the 19th century (Figure 1).

## Discussion

Our findings illustrate the broader benefits of screening DNA libraries for bycatch sequences (i.e., non-target sequences or non-host sequences). Bycatch can yield informative data that are unavailable elsewhere, such as the presence of pathogen and microbiome data. For this reason, we advise screening libraries for bycatch sequences of interest rather than discarding the data after initial target-species analyses. Even when small numbers of bycatch reads are recovered (as in 5 of our libraries), these screens provide valuable preliminary data for future studies. For instance, two Hawaii ‘amakihi dating to the mid-19th century (ANSP 30018 and ANSP 30019; Table S1) show evidence of possible *Avipoxvirus* infection (23 and 1 reads, respectively). These are ideal candidates for deeper sequencing to explore the origins of *Avipoxvirus* in the Hawaiian archipelago.

Our Hawaiian *Avipoxvirus* genome provides insight into this strain’s evolutionary relationships with other *Avipoxvirus* strains throughout the world. These Hawaiian *Avipoxvirus* strains are most closely related to extant canarypox virus, which falls into a clade with shearwaterpox virus and magpiepox virus. The strains’ similarity to canarypox virus suggests that they originated from introduced passerines during the 19th century, rather than through introduced poultry as previously assumed.^17^ This is similar to the pattern observed in the Galapagos, where *Avipoxvirus* likely derived from introduced passerines.^14^ While *Avipoxvirus* has multiple documented modes of transmission, the virus’s establishment in Hawaii was likely enhanced by the introduction of the *Culex quinquefasciatus* in the early 19th century as mosquito-vectored transmission is the predominant mode in extant Hawaiian honeycreepers.^3^^,^^6^ Notably, canarypox-like *Avipoxvirus* has become established in Hawaiian land birds, while the closely related shearwaterpox and magpiepox viruses have yet to be detected in Hawaii. The presence of migratory shearwaters and other Procellariiformes on the islands and the large number of Passeriformes introductions to Hawaii since the 19th century provide ample opportunities for these viruses’ introductions. Pox infections are well documented in Laysan albatross (*Phoebastria immutabilis*) on Oahu, although the identified causative strain identified in two chicks matched the canarypox-like strain found in Hawaiian endemic land birds.^18^ This pattern can arise from at least four scenarios: (1) shearwaterpox and magpiepox are circulating in the Hawaiian endemic land bird populations at too low a frequency to have been detected in previous *Avipoxvirus* surveys; (2) shearwaterpox and magpiepox have never reached the Hawaiian islands despite the presence of their hosts; (3) shearwaterpox and magpiepox do not readily transmit to Hawaiian land birds (e.g., due to host immunity); or (4) shearwaterpox and magpiepox are so virulent in Hawaiian endemic land birds that the hosts die before the viruses are able to transmit and become established. If some of the Laysan albatross infections are shearwaterpox, this implies either a possible transmission barrier between Hawaiian sea and land birds or a competitive advantage for the canarypox-like *Avipoxvirus* strains in Hawaii.

Since their introduction, both Hawaiian canarypox-like *Avipoxvirus* clusters have persisted in the islands for over 100 years. Notably, the 1887 case represents the earliest molecularly verified case of *Avipoxvirus* in the Hawaiian Islands, predating the previously identified 1900 case by 13 years.^2^ The 1887 ʻalalā case corresponds well with the historical observations of native bird die-offs in the late 19th century, which have frequently been attributed to *Avipoxvirus* through limited evidence.^3^^,^^6^ We provide unambiguous genetic evidence for the presence of *Avipoxvirus* on Hawaii Island by 1887, supporting the physical evidence from the 1888 ‘ō‘ū *Avipoxvirus*-like lesion. Furthermore, the ʻalalā is now extinct in the wild. Our data suggest that *Avipoxvirus* may have contributed to the ʻalalā’s decline, although we lack definitive evidence beyond the singular case of infection.

### Limitations of the study

Worldwide *Avipoxvirus* diversity has primarily been assessed using a 538 bp fragment of the *4b core protein* gene^1^^,^^2^ as no genome sequences for extant Hawaiian *Avipoxvirus* yet exist. While we now have a genome sequence for 1 of the 2 documented canarypox-like *Avipoxvirus* strains found in Hawaiian endemic birds, the second strain remains unsequenced. Further research should focus on the origins of *Avipoxviruses* in Hawaii, as well as their evolution since their introduction to the region, using whole-genome sequencing.

A secondary challenge is that the presence of *Avipoxvirus* lesions or DNA in museum specimens does not necessarily indicate significant morbidity or mortality due to the infection as extant Hawaiian endemic birds often recover from infections.^3^ Additional evidence (e.g., surveys of healed versus unhealed lesions in historic specimens and diachronic genomic surveys) is needed to infer historical epidemiology and possible co-evolution between the virus and host species over the last ∼150 years.

## Resource availability

### Lead contact

Requests for further information and resources should be directed and will be fulfilled by the lead contact, Michael G. Campana (campanam@si.edu).

### Materials availability

This study did not generate new unique reagents.

### Data and code availability

• Raw DNA sequence data have been deposited in the NCBI Sequence Read Archive (https://www.ncbi.nlm.nih.gov/sra). Phylogenetic trees and Hawaiian *Avipoxvirus* genome consensus sequences are available in the Smithsonian Figshare repository (https://smithsonian.figshare.com/).
• All original code has been deposited at GitHub (https://github.com/campanam/Avipoxvirus).
• Any additional information required to reanalyze the data reported in this paper is available from the lead contact upon request.

## Acknowledgments

This research was supported by the 10.13039/100000014Smithsonian Institution, the 10.13039/100000001National Science Foundation (DEB-1547168, DEB-1717498, and DEB-2001213), and the 10.13039/100000202United States Fish and Wildlife Service (F20AC11193-00). We thank the curators and collection managers who provided samples of museum specimens under their care (Table S1) and fieldworkers who provided blood samples.

## Author contributions

R.C.F. and M.G.C. designed the study. M.W.E.-G. and M.G.C. performed the analyses. M.W.E.-G. wrote the manuscript with input from M.G.C. All authors reviewed, edited, and approved the manuscript.

## Declaration of interests

The authors declare no competing interests.

## STAR★Methods

### Key resources table

**Table:** 

REAGENT or RESOURCE	SOURCE	IDENTIFIER
**Deposited data**		

Novel raw sequence data for six *Avipoxvirus*-positive Hawaii ‘amakihi (*Chlorodrepanis virens*)	This paper	NCBI BioProject: PRJNA1112553
Published raw sequence data from Bachman’s warblers (*Vermivora bachmanii*)	Byerly et al.^19^	NCBI BioProject: PRJNA994867
Published raw sequence data from ‘akikiki (*Oreomystis bairdi*) and ‘akeke‘e (*Loxops caeruleirostris*)	Cassin-Sackett et al.^20^	NCBI BioProject: PRJNA527134
Published raw sequence data from Hawaii ‘amakihi (*Chlorodrepanis virens*)	Cassin-Sackett et al.^21^	https://doi.org/10.1111/mec.14891
Published raw sequence data from ‘alalā (*Corvus hawaiiensis*)	Blanchet et al.^22^	NCBI BioProject: PRJNA975161
Publicly available *Avipoxvirus* whole genome sequences	GenBank	GenBank: AF198100.1, AJ581527.1, AY318871.1, CQ970871.1, DD211553.1, FW576866.1, KJ801920.1, KJ859677.1, KP728110.2, KX196452.1, KX857215.1, KX857216.1, MF678796.1, MF766430.1, MF766431.1, MF766432.1, MG702259.1, MG760432.1, MH709124.1, MH709125.1, MH719203.1, MH734528.1, MK903864.1, MT978051.1, MW142017.1, MW296038.1, MW365933.1, MW485973.1, MW558065.1, MW558066.1, MW558067.1, MW558068.1, MW558069.1, MW558070.1, MW558071.1, MW558072.1, MW558073.1, MW558074.1, MW558075.1, MW558076.1, MW558077.1, MW558078.1, MW558079.1, MW558080.1, MW558081.1, NC_002188.1, NC_005309.1, NC_024446.1, NC_024447.1, NC_036582.1, OK345039.1, OK345040.1, OK348853.1, OK558608.1, OK558609.1, ON375849.1, ON408417.1.
Publicly available *Avipoxvirus* conserved gene sequences	GenBank	See Table S2
Phylogenetic trees and Hawaiian *Avipoxvirus* genome consensus sequences	This paper	https://doi.org/10.25573/data.25833037

**Software and algorithms**		

*Avipoxvirus* analysis pipeline v. 0.1.0	This paper	https://github.com/campanam/Avipoxvirus https://doi.org/10.5281/zenodo.14010732
Nextflow v. 22.04.4	Di Tommaso et al.^23^	https://nextflow.io/
AdapterRemoval v. 2.3.3	Schubert et al.^24^	https://github.com/MikkelSchubert/adapterremoval
Seqtk v. 1.4	H. Li	https://github.com/lh3/seqtk
CD-HIT v. 4.8.1	Li and Godzik^25^	https://www.bioinformatics.org/cd-hit/
BLAST+ v. 2.13.0	Camacho et al.^26^	https://ftp.ncbi.nlm.nih.gov/blast/executables/blast+/LATEST/
MEGAN6-CE v. 6.24.20	Huson et al.^27^	https://software-ab.cs.uni-tuebingen.de/download/megan6/welcome.html
BWA v.0.7.17-r1188	Li and Durbin^28^	https://bio-bwa.sourceforge.net/
SAMtools v. 1.18	Danecek et al.^29^	https://www.htslib.org/
Genome Analysis Toolkit v. 4.4.0.0	McKenna et al.^30^	https://gatk.broadinstitute.org/hc/en-us
DamageProfiler v. 1.1	Neukamm et al.^31^	https://github.com/Integrative-Transcriptomics/DamageProfiler
Geneious Prime v. 2023.0.4	Biomatters, Ltd.	https://www.geneious.com/
Bowtie 2 Geneious plugin v. 7.2.2	Langmead and Salzberg^32^	https://bowtie-bio.sourceforge.net/bowtie2/index.shtml; https://www.geneious.com/plugins
MAFFT v. 7.490 Geneious plugin	Katoh and Standley^33^	https://mafft.cbrc.jp/alignment/server/index.html; https://www.geneious.com/plugins
RAxML v. 8.2.11 Geneious plugin	Stamatakis^34^	https://cme.h-its.org/exelixis/web/software/raxml/; https://www.geneious.com/plugins

### Experimental model and study participant details

Our dataset consisted of 719 (of which 639 derived from Hawaiian birds) sequencing libraries derived from blood (n = 331) and museum tissue (n = 178) unique samples. Some individuals (n = 198) were sequenced multiple times. Libraries consisted of whole-genome shotgun sequencing libraries (n = 9) and targeted sequencing libraries (n = 710) using various myBaits (Daicel Arbor Biosciences) capture assays. We analyzed 209 Hawaii ‘amakihi (*Chlorodrepanis virens*), 71 Oahu ‘amakihi (*Chlorodrepanis flava*), 20 ‘alalā (*Corvus hawaiiensis*), 64 ‘akikiki (*Oreomystis bairdi*), 37 ‘akeke‘e (*Loxops caeruleirostris*) and 39 samples from other species found on the Hawaiian Islands. As a control, we screened 33 Bachman’s warblers (*Vermivora bachmanii*) museum specimens^11^ and another 36 blood samples from non-Hawaiian birds. We processed 10 extraction and library preparation negative controls (blanks containing extraction/library preparation reagents but no DNA sample) to ensure the reliability of our results. Data derived from published studies^19^^,^^20^^,^^21^^,^^22^ and in-preparation manuscripts from the Center for Conservation Genomics (Henschen, Kearns, vanTassel, Campana, Fleischer et al., unpublished data). Museum specimens were processed in specialist ancient DNA laboratories separated from modern samples according to standard ancient DNA protocols (e.g.,^19^^,^^35^).

### Method details

#### Avipoxvirus screening pipeline

We screened each bidirectionally sequenced Illumina library using a custom Nextflow^23^ domain-specific language (DSL) 2 pipeline (https://github.com/campanam/Avipoxvirus). We removed adapter sequences and merged forward and reverse reads using AdapterRemoval v. 2.3.3^24^ with the options ‘--collapse --gzip --minlength 30’. We converted the merged sequences from FASTQ to FASTA format using Seqtk v. 1.4 (https://github.com/lh3/seqtk) and removed polymerase chain reaction (PCR) duplicate sequences with cd-hit-est (from CD-HIT v. 4.8.1^25^) with the options ‘-c 1 -M 0’.

Next, we constructed a custom BLAST+ v. 2.13.0^26^ database of complete *Avipoxvirus* genome sequences available in the National Center for Biotechnology Information (NCBI) GenBank database. Our database included the following accessions: GenBank: AF198100.1, AJ581527.1, AY318871.1, CQ970871.1, DD211553.1, FW576866.1, KJ801920.1, KJ859677.1, KP728110.2, KX196452.1, KX857215.1, KX857216.1, MF678796.1, MF766430.1, MF766431.1, MF766432.1, MG702259.1, MG760432.1, MH709124.1, MH709125.1, MH719203.1, MH734528.1, MK903864.1, MT978051.1, MW142017.1, MW296038.1, MW365933.1, MW485973.1, MW558065.1, MW558066.1, MW558067.1, MW558068.1, MW558069.1, MW558070.1, MW558071.1, MW558072.1, MW558073.1, MW558074.1, MW558075.1, MW558076.1, MW558077.1, MW558078.1, MW558079.1, MW558080.1, MW558081.1, NC_002188.1, NC_005309.1, NC_024446.1, NC_024447.1, NC_036582.1, OK345039.1, OK345040.1, OK348853.1, OK558608.1, OK558609.1, ON375849.1, ON408417.1.

We used megaBLAST (from BLAST+ v. 2.13.0) under default parameters to compare the processed sequence data against the custom *Avipoxvirus* database, outputting the results in XML format (option ‘-outfmt 5’), and converted the megaBLAST results to RMA6 and LCA formats using the blast2rma (option ‘-supp 0’) and blast2lca scripts from MEGAN6-CE v. 6.24.20.^27^ We then extracted reads that matched the Bamfordvirae taxon using the read-extractor script from MEGAN6-CE and generated a CSV-format table summarizing the number of Bamfordvirae-assigned reads per library. We then compared the Bamfordvirae-assigned reads assigned against NCBI’s complete non-redundant nucleotide database (database ‘nt’, dated July 7, 2022) and generated a second summary table of hits. This step eliminated any hits that may have aligned more closely to data from species outside the Bamfordvirae were assigned to the taxon due to the initially limited BLAST+ database.

#### DNA damage profiling

Two specimens (USNM 169333 and CamMZ-27/Cor/5/gg/8) yielded enough *Avipoxvirus* sequences for DNA damage profiling. We aligned the identified *Avipoxvirus* reads against the Canarypox virus reference genome^11^ (GenBank: NC_005309) using BWA-ALN v. 0.7.17-r1188^28^ disabling the alignment seed (option ‘-l 1024’) following Schubert et al.^36^ We fixed mate-pair tags and sorted the alignments using SAMtools v. 1.18,^29^ left-aligned indels using the Genome Analysis Toolkit v. 4.4.0.0,^30^ and marked duplicates using SAMtools. We then profiled DNA fragmentation and cytosine deamination using DamageProfiler v. 1.1^31^ (Figures S1 and S2).

#### Bowtie 2 alignment against reference genomes

Using the Bowtie 2^32^ plugin (v. 7.2.2) in Geneious Prime v. 2023.0.4 (https://www.geneious.com/), we aligned the quality-controlled, deduplicated USNM 169333 and CamMZ-27/Cor/5/gg/8 *Avipoxvirus* reads against each reference genome using the preset parameters ‘High Sensitivity / Medium’ and the ‘Local’ alignment type. We did not trim reads before mapping. We set the maximum mismatches to 0, the seed length to 22, and the minimum and maximum insert lengths to 0 and 800 respectively. We reported only the best alignment match. We exported the alignment in binary alignment map (BAM) format and calculated sequencing coverage and depth using the SAMtools v. 1.18^29^ ‘coverage’ command.

We generated a consensus sequence in Geneious using the ‘Highest Quality (60%)’ threshold, a 65% threshold of sequences without quality and assigned quality using the ‘Total’ option. For consensus sequences, we required a minimum coverage of 2 (otherwise the base was called as ‘?’) and called Sanger heterozygotes for bases with more than 50% different bases. We then aligned the USNM 169333 *Avipoxvirus* consensus sequence against each reference genome using the MAFFT v. 7.490^33^ plugin in Geneious Prime under default parameters (Algorithm: ‘Auto’; Scoring Matrix: ‘200PAM / k=2’; Gap open penalty: 1.53; Offset value: 0.123). Sequence identity statistics were then calculated using Geneious Prime.

#### Conserved *Avipoxvirus* genes

To be consistent with previous historic Hawaiian *Avipoxvirus* research, we downloaded 82 *4b* DNA sequences that matched the region specified in Jarvi et al.^2^ (Table S2). We extracted the *4b* region from our USNM 169333 reads by aligning the reads to the Canarypox virus *4b* region (GenBank: GU108510.1) using the Bowtie 2 plugin and generating the consensus sequence as above. We then aligned the USNM 169333 consensus sequence and the GenBank sequences using the MAFFT plugin and the Geneious consensus aligner under default settings. We then manually trimmed the aligned sequences to the target 538 bp region. We used the RAxML v. 8.2.11^34^ plugin in Geneious Prime to build a maximum likelihood tree with 1000 bootstrap replicates (options ‘-m GTRGAMMA -f a -x 1 -N 1000 -p 234233 -k). To place the Hawaiian strains in context of their worldwide diversity, we repeated this analysis using an extended dataset of 529 *4b* sequences from GenBank. We included all *4b* sequences with a pre-alignment-trimming length of at least 500 bp that we could confidently align to the 538 bp sequence. The final RAxML trees are available in the Smithsonian Figshare repository (https://doi.org/10.25573/data.25833037).

Following Sarker et al.,^16^ we analyzed 8 conserved *Avipoxvirus* protein genes frequently used in phylogenetic classification: *DNA polymerase*, *mRNA capping enzyme large subunit*, *NTPase*, *P4a*, *RAP94*, *RPO132*, *RPO147*, and *VETFL*. The majority of available *Avipoxvirus* data are limited to protein sequences, so we downloaded amino acid sequences corresponding to each gene from GenBank (Table S2). We obtained 55 *DNA polymerase*, 51 *mRNA capping enzyme large subunit*, 48 *NTPase*, 51 *P4a*, 55 *RAP94*, 50 *RPO132*, 50 *RPO147*, and 26 *VETFL* sequences.

For each gene, we extracted the corresponding annotated DNA sequence from the Canarypox virus reference genome (GenBank: NC_005309.1). We then aligned the USNM 169333 and CamMZ-27/Cor/5/gg/8 reads against the corresponding sequence using Bowtie 2 and called a consensus sequence as above. After manual trimming, we translated the USNM 169333 sequence into amino acids using Geneious Prime. We then aligned the translated sequences and downloaded reference sequences using MAFFT v. 7.490 as above except that we set the algorithm to ‘L-INS-I’ and the scoring matrix to ‘BLOSUM62’ (based on the settings in Sarker et al.^16^). After alignment, we built RAxML maximum likelihood trees with 1000 bootstrap replicates as above, but adjusting the model for amino acid data (options ‘-m PROTGAMMABLOSUM62 -f a -x 1 -N 1000 -p 234233 -k’). The final RAxML trees are available in the Smithsonian Figshare repository (https://doi.org/10.25573/data.25833037).

### Quantification and statistical analysis

DNA alignment and composition statistics are provided in the results section. These statistics were calculated using the custom Nextflow pipeline described above and Geneious Prime. DNA damage profiles produced by DamageProfiler are presented in Figures S1 and S2. For each gene tree topology, maximum likelihood tree clade support was evaluated using 1000 bootstrap replicates. Clades’ percent bootstrap support values are provided in the phylogenetic trees included in the Smithsonian Figshare repository and in Figure 1.
