# Supplementary material for: A historical Hawaiian Avipoxvirus genome reconstructed from an 1898 museum specimen
Source: iScience. 2025 Mar 3;28(4):112153. doi: 10.1016/j.isci.2025.112153 (PMC12131269; doi:10.1016/j.isci.2025.112153)
Supplement: Document S1. Figures S1 and S2 and Tables S1 and S2 [file mmc1.pdf]

iScience, Volume *28*

Supplemental information

A historical Hawaiian *Avipoxvirus* genome reconstructed from an 1898 museum specimen

Madeline W. Eibner-Gebhardt, Robert C. Fleischer, and Michael G. Campana

Table S1. *Avipoxvirus*-positive Museum Specimens, Related to STAR Methods.
Provenance of museum specimens yielding *Avipoxvirus* sequences. AMNH=American Museum of Natural History (New York, NY, USA); ANSP=Academy of Natural Sciences of Drexel University (Philadelphia, PA, USA); CamMZ=Cambridge University Museum of Zoology (Cambridge, UK); MVZ=Museum of Vertebrate Zoology (Berkeley, CA, USA); NMNH=National Museum of Natural History (Washington, DC, USA).

**Accession**	**Museum**	**Date**	**Provenance**	**Collector**	***Avipoxvirus* Reads**	**Species**	**NCBI Accession**
ANSP 30018	ANSP	1850s–1860s	Hawaii	Andrew Garrett	23	*Chlorodrepanis virens*	SAMN41430680
ANSP 30019	ANSP	1850s–1860s	Hawaii	Andrew Garrett	1	*Chlorodrepanis virens*	SAMN41430681
AMNH 453111	AMNH	1888	Waimea, Hawaii	Scott B. Wilson	1	*Chlorodrepanis virens*	SAMN41430682
MVZ:Bird:21477	MVZ	1903	Homer’s Ranch, Hamakua, Hawaii	Chester E. Blacow	15	*Chlorodrepanis virens*	SAMN41430683
MVZ:Bird:118734	MVZ	1948	Kipuka Papalinamoku, Kau, Hawaii	Paul H. Baldwin	7	*Chlorodrepanis virens*	SAMN41430684
USNM 169333	NMNH	1898	Kaumana, Hawaii	Henry W. Henshaw	27,569	*Chlorodrepanis virens*	SAMN41430685
CamMZ-27/Cor/5/gg/8	CamMZ	1887	Kaawaloa, Kona, Hawaii	Scott B. Wilson	1,157	*Corvus hawaiiensis*	SAMN35311562

Table S2. GenBank Accessions for Conserved *Avipoxvirus* Genes, Related to STAR Methods.

Gene	Accessions
4b core protein (538 bp dataset)	AB292647.1, AY530302.1, AY530304.1, AY530307.1–AY530311.1, EF568377.1–EF568401.1, GU108502.1, GU108510.1, MH175220.1–MH175224.1, MH175226.1, MH175228.1, MH175250.1–MH175255.1, MH175257.1–MH175261.1, MH175264.1–MH175265.1, MH175267.1–MH175284.1, MH175286.1–MH175292.1, MH175294.1–MH175295.1
4b core protein (extended dataset)	AB292647.1, AB576861.1, AM050375.1–AM050380.1, AM050382.1–AM050392.1, AY530302.1–AY530311.1, DQ131891.1–DQ131902.1, DQ873808.1–DQ873811.1, EF016108.1, EF568377.1–EF568401.1, EU798994.1–EU798995.1, FJ863095.1–FJ863096.1, FR852585.1–FR852586.1, GQ180200.1–GQ180213.1, GQ221269.1, GQ487567.1, GU108500.1–GU108510.1, HM623675.1, HQ441566.1, HQ875129.1–HQ875130.1, JN615018.1, JQ665838.1–JQ665840.1, JX418296.1, JX464819.1–JX464821.1, KC017960.1–KC017962.1, KC017964.1–KC017970.1, KC017972.1–KC018037.1, KC018039.1–KC018069.1, KC193679.1, KC588955.1–KC588961.1, KF032407.1, KF548036.1–KF548037.1, KF673397.1, KF875986.1, KF956000.1–KF956003.1, KJ192189.1–KJ192191.1, KJ809101.1, KR297259.1–KR297262.1, KR733092.1, KT003288.1–KT003290.1, KT343757.1, KU212807.1, KU522209.1–KU522210.1, KX774282.1–KX774286.1, KY569404.1, LC055558.1–LC055564.1, LC481450.1–LC481451.1, LC499632.1, LC545571.1–LC545572.1, LC796261.1–LC796265.1, M25781.1, MF102266.1–MF102272.1, MF140450.1, MG601779.1–MG601782.1, MG934698.1, MH106510.1, MH175215.1–MH175296.1, MH286510.1, MK651852.1–MK651861.1, MN257631.1, MN915017.1, MN939477.1, MN971579.1, MT332851.1, MT756471.1, MT877435.1–MT877440.1, MT929367.1, MT929368.1, MW147459.1, MW147745.1, MW349699.1, MW349701.1, MW602950.1, MZ091383.1–MZ091393.1, MZ409513.1, MZ614735.1–MZ614742.1, MZ706997.1–MZ707002.1, NC_043178.1, OK483026.1–OK483028.1, OL634783.1–OL634795.1, OL703782.1–OL703784.1, OL703786.1–OL703791.1, OL830428.1–OL830432.1, OM364080.1, ON568594.1–ON568595.1, ON568598.1–ON568602.1, ON568605.1, ON568610.1–ON568613.1, ON568617.1–ON568618.1, ON568620.1, ON568623.1, ON568629.1, ON568631.1–ON568635.1, ON568637.1, OP039560.1, OP131512.1–OP131515.1, OQ064751.1–OQ064753.1, OQ077515.1–OQ077517.1, OQ615872.1–OQ615881.1, OR920788.1–OR920789.1, OR988064.1–OR988067.1, PP481179.1, PP502427.1, PP756527.1, PQ038259.1, PQ202258.1–PQ202262.1, PQ497102.1–PQ497103.1, PQ580322.1–PQ580325.1
DNA polymerase	1717319A, AAA43821, AAR83467, AF198100_85, AID46604, AID46833, ALA62442, ALK24309, ART91528, AUD40198, AWD84597, AXY04536, AXY04798, AXY05057, AYO89688, AYO89947, AYO90206, AYO90463, AYP74207, CAA11286, CAE52636, NP_039057, NP_955144, P21402, QGM48753, QRM13631, QZW33423, UHJ14922, UHJ15179, UPX08851, UPX08852–UPX08853, UQT20389, UQT20630, URH24813, URH25072, URH25336, URH25596, URH25855, URH26382, URH26644, URH26909, URH27171, URH27435, URH27699, URH27960, URH28219, URH28478, URH28737, URH28996, WCL40045, YP_009046091, YP_009046328, YP_009177089, YP_009448013
mRNA capping enzyme large subunit	AAR83538, AF198100_137, AID46653, AID46882, ALA62484, ARE67418, ARF02744, ART91579, AUD40250, AWD84668, AXY04588, AXY04850, AXY05109, AYO89739, AYO89998, AYO90257, AYO90514, AYP74153, CAE52685, NP_039109, NP_955215, Q9J584, QGM48824, QRM13686, QZW33508, UHJ14924, UHJ15181, UQT20441, UQT20685, URH24865, URH25124, URH25388, URH25648, URH25907, URH26171, URH26434, URH26696, URH26961, URH27223, URH27487, URH27751, URH28012, URH28269, URH28528, URH28787, URH29048, WCL40094, YP_009046140, YP_009046377, YP_009177131, YP_009448065
NTPase	AAR83428, AF198100_49, AID46571, AID46800, ALA62409, ARE67300, ARF02677, AUD40160, AWD84558, AXY04500, AXY04762, AXY05021, AYO89653, AYO89912, AYO90171, AYO90428, AYP74239, CAE52603, NP_039021, NP_955105, QGM48712, QRM13595, QZW33379, UHJ14927, UHJ15184, UQT20353, UQT20594, URH24776, URH25034, URH25298, URH25559, URH25817, URH26344, URH26606, URH26871, URH27133, URH27397, URH27661, URH27923, URH28179, URH28438, URH28697, URH28957, WCL40012, YP_009046058, YP_009046295, YP_009177056, YP_009447975
P4a	AAR83593, AF198100_165, AID46681, AID46912, ALA62502, ARE67484, ARF02790, ART91607, AUD40281, AWD84723, AXY04616, AXY04878, AXY05137, AYO89767, AYO90026, AYO90285,

	AYO90542, AYP74121, CAE52711, NP_039137, NP_955270, Q9J559, QGM48880, QRM13719, QZW33578, UHJ14923, UHJ15180, UQT20472, UQT20716, URH24895, URH25155, URH25418, URH25678, URH25937, URH26201, URH26464, URH26726, URH26992, URH27254, URH27517, URH27782, URH28043, URH28298, URH28557, URH28816, URH29079, WCL40122, YP_009046170, YP_009046405, YP_009177149, YP_009448096
RAP94	2209386A, AAR83533, AF198100_132, AHL69635, AID46648, AID46877, ALA62479, ARE67411, ARF02741, ART91574, AUD40245, AWD84663, AWV49179, AXY04583, AXY04845, AXY05104, AYO89734, AYO89993, AYO90252, AYO90509, AYP74158, CAE52680, L46396_1, NP_039104, NP_955210, Q9J589, QGM48819, QRM13681, QZW33502, UHJ14926, UHJ15183, UQT20436, UQT20680, URH24860, URH25119, URH25383, URH25643, URH25902, URH26166, URH26429, URH26691, URH26956, URH27218, URH27482, URH27746, URH28007, URH28264, URH28523, URH28782, URH29043, WCL40089, YP_009046135, YP_009046372, YP_009177126, YP_009448060
RPO132	AAR83609, AF198100_180, AID46696, AID46928, ALA62513, ARE67500, ARF02800, ART91622, AUD40297, AWD84739, AXY04631, AXY04893, AXY05152, AYO89782, AYO90041, AYO90300, AYO90557, AYP74106, CAE52727, NP_039152, NP_955286, Q9J544, QGM48897, QRM13735, QZW33594, UHJ14921, UHJ15178, UQT20488, UQT20732, URH24908, URH25170, URH25433, URH25691, URH25952, URH26479, URH26741, URH27007, URH27269, URH27532, URH27798, URH28056, URH28311, URH28571, URH28829, URH29095, WCL40137, YP_009046186, YP_009046420, YP_009177160, YP_009448112
RPO147	AAR83528, AF198100_128, AID46644, AID46873, ALK24310, ARE67406, ARF02736, ART91570, AUD40241, AWD84658, AXY04579, AXY04841, AXY05100, AYO89730, AYO89989, AYO90248, AYO90505, AYP74162, CAE52676, NP_039100, NP_955205, Q9J593, QGM48813, QRM13677, QZW33496, UHJ14920, UHJ15177, UQT20432, UQT20676, URH24856, URH25115, URH25379, URH25639, URH25898, URH26162, URH26425, URH26687, URH26952, URH27214, URH27478, URH27742, URH28003, URH28260, URH28519, URH28778, URH29039, WCL40085, YP_009046131, YP_009046368, YP_009448056
VETFL	AAR83590, ARE67481, ARF02788, AWD84720, AYP74124, ETF2_FOWPN, NP_955267, QGM48877, QZW33575, URH24892, URH25152, URH25415, URH25675, URH25934, URH26198, URH26461, URH26723, URH26989, URH27251, URH27514, URH27779, URH28040, URH28295, URH28554, URH28813, URH29076

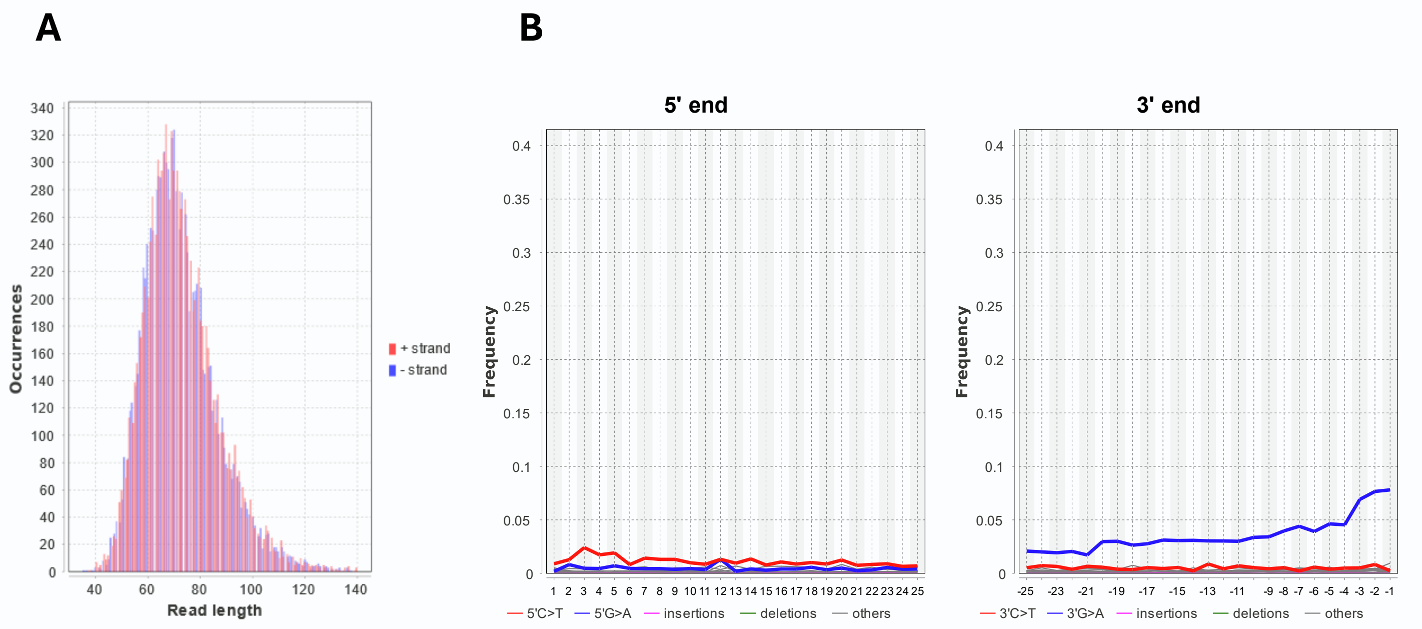

Figure S1: DNA fragmentation (A) and cytosine deamination (B) profiles for specimen USNM 169333, Related to STAR Methods.

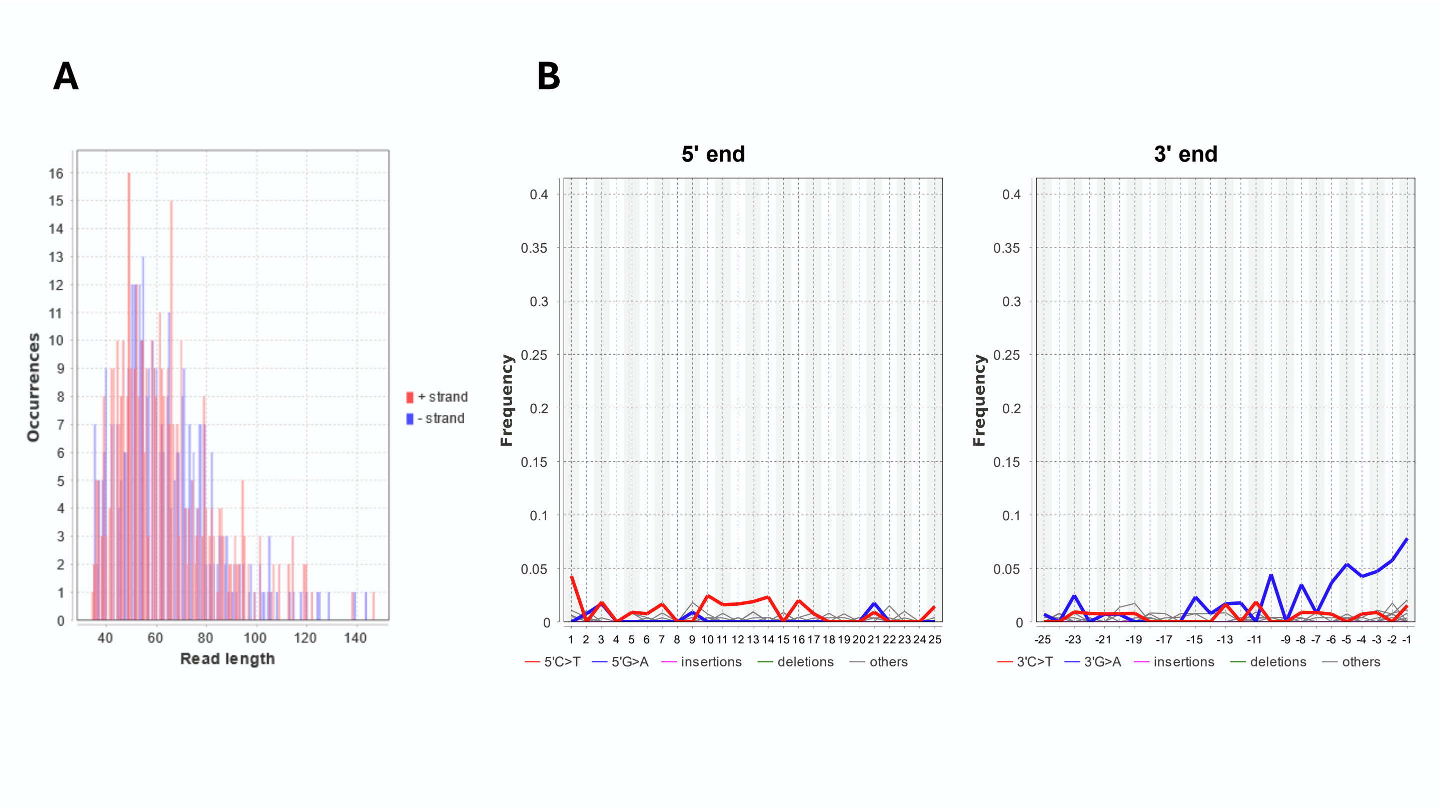

Figure S2: DNA fragmentation (A) and cytosine deamination (B) profiles for specimen CamMZ-27/Cor/5/gg/8, Related to STAR Methods.
